# Supplementary material for: Cyclic AMP compartmentalization drives signal specificity to control vector colonization and mammalian host infection by American trypanosomes
Source: PLoS Pathog. 2026 Jun 29;22(6):e1013784. doi: 10.1371/journal.ppat.1013784 (PMC13340794; doi:10.1371/journal.ppat.1013784)
Supplement: S1 Table — (DOCX) [file ppat.1013784.s006.docx]

**Table S1.** Gene IDs of PDE orthologs used for the phylogenetic analysis of Figure 1E.

| **PDEA** | **Organism/strain** | **Sequence ID*** |
| --- | --- | --- |
|  | *Trypanosoma cruzi* Y C6 | TcYC6_0116440 |
|  | *Bodo saltans* strain Lake Konstanz | BSAL_07345 |
|  | *Crithidia fasciculata* strain Cf-Cl | CFAC1_140020300 |
|  | *Leishmania donovani* CL-SL | LdCL_180016200 |
|  | *Leishmania braziliensis* MHOM/BR/75/M2904 | LbrM.18.1190 |
|  | *Leishmania mexicana* MHOM/GT/2001/U1103 | LmxM.18.1090 |
|  | *Paratrypanosoma confusum* CUL13 | PCON_0060640 |
|  | *Trypanosoma brucei brucei* TREU927 | Tb927.10.13000 |
| **PDED** | *Trypanosoma cruzi* Y C6 | TcYC6_0110850 |
|  | *Bodo saltans* strain Lake Konstanz | BSAL_59000 |
|  | *Crithidia fasciculata* strain Cf-Cl | CFAC1_200032200 |
|  | *Leishmania donovani* CL-SL | LdCL_290031600 |
|  | *Leishmania braziliensis* MHOM/BR/75/M2904 | LbrM.29.2410 |
|  | *Leishmania mexicana* MHOM/GT/2001/U1103 | LmxM.08_29.2440 |
|  | *Paratrypanosoma confusum* CUL13 | PCON_0039780 |
|  | *Trypanosoma brucei brucei* TREU927 | Tb927.3.3340 |
| **PDEB1** | *Trypanosoma cruzi* Y C6 | TcYC6_0028510 |
|  | *Bodo saltans* strain Lake Konstanz | BSAL_27845 |
|  | *Crithidia fasciculata* strain Cf-Cl | CFAC1_240048400 |
|  | *Leishmania donovani* CL-SL | LdCL_150020700 |
|  | *Leishmania braziliensis* MHOM/BR/75/M2904 | LbrM.15.1480 |
|  | *Leishmania mexicana* MHOM/GT/2001/U1103 | LmxM.15.1481 |
|  | *Paratrypanosoma confusum* CUL13 | **No ortholog** |
|  | *Trypanosoma brucei brucei* TREU927 | Tb927.9.5040 |
| **PDEB2** | *Trypanosoma cruzi* Y C6 | TcYC6_0028500 |
|  | *Bodo saltans* strain Lake Konstanz | **No ortholog** |
|  | *Crithidia fasciculata* strain Cf-Cl | CFAC1_240048300 |
|  | *Leishmania donovani* CL-SL | LdCL_150020800 |
|  | *Leishmania braziliensis* MHOM/BR/75/M2904 | LbrM.15.1250 |
|  | *Leishmania mexicana* MHOM/GT/2001/U1103 | LmxM.15.1480 |
|  | *Paratrypanosoma confusum* CUL13 | **No ortholog** |
|  | *Trypanosoma brucei brucei* TREU927 | Tb927.9.5100 |
| **PDEC** | *Trypanosoma cruzi* Y C6 | TcYC6_0111120 |
|  | *Bodo saltans* strain Lake Konstanz | **No ortholog** |
|  | *Crithidia fasciculata* strain Cf-Cl | CFAC1_200034700 |
|  | *Leishmania donovani* CL-SL | LdCL_290034000 |
|  | *Leishmania braziliensis* MHOM/BR/75/M2904 | LbrM.29.2740 |
|  | *Leishmania mexicana* MHOM/GT/2001/U1103 | LmxM.08_29.2680 |
|  | *Paratrypanosoma confusum* CUL13 | PCON_0039560 |
|  | *Trypanosoma brucei brucei* TREU927 | Tb927.3.3070 |
| **PDE4A isoform 5** | Homo sapiens | NP 001230050.1** |

* Gene IDs retrieved from TriTrypDB

** NCBI Reference Sequence
