# Supplementary material for: Cyclic AMP compartmentalization drives signal specificity to control vector colonization and mammalian host infection by American trypanosomes
Source: PLoS Pathog. 2026 Jun 29;22(6):e1013784. doi: 10.1371/journal.ppat.1013784 (PMC13340794; doi:10.1371/journal.ppat.1013784)
Supplement: S2 Table — (DOCX) [file ppat.1013784.s007.docx]

**Table S2**: List of oligonucleotides used in this study.

| **N^0^** | **Primer name** | **Sequence (5’→3’)** |
| --- | --- | --- |
| 1 | RvG00 | AAAAGCACCGACGGTGCCACTTTTAAGTTGATAACGGACTAGCCTTATTTT AACTTGCTATTTAGCTAAAAC |
| 2 | FwPDEC_sgRNA_KO | GAAATTAATACGACACTATAGG**GAAATGCTCAGGGGTTAG**GTTTTAGAGCTAGAAATAGC |
| 3 | FwPDEC_KO | GAACGAGAGAGAAGTTTGGGAGTTCCTTTGG CGTGCCGCGGGAATGATTATG |
| 4 | RvPDEC_KO | CGCAGCCATAAACTTTTAGCACTGCGAACA GCGCGAATACTAGTGATTAC |
| 5 | FwPDEC_KO-chk_ext | GAAGGATAAGAATGCGTGG |
| 6 | RvPDEC_KO-chk_ext | CAATAGCAGCCGATATAACGC |
| 7 | FwPDEC_KO-chk_int | GGAAGAAGTTGCGACT |
| 8 | RvPDEC_KO-chk_int | CCAAATGGCTTTGCAG |
| 9 | FwPDEB1/2_sgRNA_KO | GAAATTAATACGACACTATAGG**GACGTACGAGGGGGATTG**GTTTTAGAGCTAGAAATAGC |
| 10 | FwPDEB1/2_KO | GAGGCAACTTGGCAACCAATGTTTACAACAACGCCTGCCGCGGGAATGATTATG |
| 11 | RvPDEB1/2_KO | ATTGAAATTATATATACAATTTATTCACG CGAATACTAGTGATTAC |
| 12 | FwPDEB1/2_KO-chk_ext | GCTGCATGATTGAA |
| 13 | RvPDEB1/2_KO-chk_ext | GAGGGTGAAAGAAACAAG |
| 14 | FwPDEB1/2_KO-chk_igr | GTTTGTGTACAGCCTGAA |
| 15 | RvPDEB1/2_KO-chk_igr | GCATTGTATATGGC |
| 16 | FwATG_Puro | ATGACCGAGTACAAGCCCAC |
| 17 | FwATG_BSD | ATGGCCAAGCCTTTGAA |
| 18 | FwPDEB1_XbaI | GA*TAGA*ATGGCGGAGACAGGCGG |
| 19 | RvPDEB1_XhoI | GA*CGAG*TTTATTCAACCGGCAGCC |
| 20 | FwPDEB2_XbaI | GAGC*TAGA*ATGTTTACAACAACGCCTGC |
| 21 | RvPDEB2_XhoI | TAA*GAG*CTTGGCGAAGTGAGCT |
| 22 | FwPDEC_XbaI | CTAGTAGAATGGGAGGACGCTGGG |
| 23 | RvPDEC_XhoI | GACGAGGCACTGCGAACAGAGTG |
| 24 | FwPDEC_sgRNA_ctag | GAAATTAATACGACACTATAGG**CGCAGTGCTGAAAAAGTTTA**GTTTTAGAGCTAGAAATAGC |
| 25 | FwPDEC_ctag_23T | TTAGTTGATGAGCCACCACTGTTGACGCAGTGCGGTACCGGGCCCCCCCGAG |
| 26 | RvPDEC_ctag_23T | CTAGAAGAACAAAACAAAACGCAGCCATAATGGCGGCCGCTAGAACTAGTGGAT |
| 27 | FwPDEC_ctag-chk | GAACGACGTAGGCATTTG |
| 28 | RvPDEC_ctag-chk | CAATAGCAGCCGATATAACGC |
| 29 | FwPDEA_sgRNA_ctag | GAAATTAATACGACACTATAGG**ATGGACAGGGATGAGAGT**GTTTTAGAGCTAGAAATAGC |
| 30 | FwPDEA_ctag_23T | GAGAGCTGCAAGGCATATTGGCTAAAATGGACAG GGAGGTACCGGGCCCCCCCGAG |
| 31 | RvPDEA_ctag_23T | CGCCCTTTGGCAAAAAACAACTTGGCGGCCGCTAGAACTAGTGGAT |
| 32 | FwPDEA_ctag-chk | GCGTTTATGGACCGCATG |
| 33 | RvPDEA_ctag-chk | CATAATGACGGGGGTGAAGAC |
| 34 | FwPDED_sgRNA_ctag | GAAATTAATACGACACTATAGG**TTATTGAGGAACATACTTGA**GTTTTAGAGCTAGAAATAGC |
| 35 | FwPDED_ctag_23T | AATAAAGACGCATGGCAGAGTTTTATTGAGGAACATACTGGTACCGGGCCCCCCCGAG |
| 36 | RvPDED_ctag_23T | TGGAGATTGTACCACGGCCTTTTTTACAGCCTTGGCGGCCGCTAGAACTAGTGGAT |
| 37 | FwPDED_ctag-chk | GCGTTTATGGACCGCATG |
| 38 | RvPDED_ctag-chk | CATAATGACGGGGGTGAAGAC |

***Bold** font: specific protospacer; underlined text: gene-specific homologous region in primers used to amplify DNA donor cassettes; *text in italics*: restriction site.
