# Supplementary material for: Cyclic AMP compartmentalization drives signal specificity to control vector colonization and mammalian host infection by American trypanosomes
Source: PLoS Pathog. 2026 Jun 29;22(6):e1013784. doi: 10.1371/journal.ppat.1013784 (PMC13340794; doi:10.1371/journal.ppat.1013784)
Supplement: Attachment — Submitted filename: Ahmed et al_Response to Reviewers.pdf [file ppat.1013784.s009.pdf]

**Response to Reviewers**

Note: Responses to reviewers are shown in blue font.

**General considerations**

Hereby, we are providing a detailed response to each one of the reviewers' constructive critiques, that have helped us to improve the manuscript. In addition to addressing the reviewers' comments, we have updated some figures to improve data presentation. Specifically, the western blot panels for PDEB1 and PDEB2 overexpression (Fig. 1C) and for PDEC-AB (Fig. 3G) were repeated to improve image quality. We also corrected the bar chart of relative cAMP content shown in Fig. 2I, after identifying an error in the analysis of this specific dataset. We therefore updated the corresponding figure. The new Figure 2I still shows significant differences in the relative cAMP content of PDEC-KO compared to the control and addback parasites. These minor corrections did not alter the interpretation of our results or the overall conclusions of the study but improved the general quality of the data.

**Reviewer #1**

**Summary:** This manuscript investigates the contribution of localised cAMP levels to *T. cruzi* growth, development, resistance to osmotic stress and vector infection by perturbing the levels of PDEs that have specific cellular location in the parasite. The experiments are well described and good quality, with good use of knockout, addback and overexpression approaches to confirm or explore in more detail observed phenotypes. We only have a few comments that the authors could consider.

We thank the reviewer for the positive evaluation and for the constructive suggestions that have helped us to improve the manuscript.

**Major Issues:**

1. Mainly, as highlighted by the authors in their discussion, the cAMP measurements are whole cell assays, while the PDE KO effects are expected to be local. Although local cAMP concentrations (dependent on local PDE levels) likely explain the phenotypes, it is also possible that there is a calibrated response at the whole cell level, with observed and different phenotypes dependent on the absolute cellular cAMP level. As an example, a growth effect is attributed to the CV PDEC acting locally on cAMP levels, but it could also be a response to overall cellular cAMP levels, which is dominantly controlled by PDE1C. As the authors suggest, local cAMP concentrations could be directly assayed by FRET based approaches (or other reporters) but I don't think this is necessary in this manuscript. However, the possibility that whole cell cAMP levels (with different phenotypes manifest at different absolute levels) is worth discussing in the manuscript.

We thank the reviewer for this important point. As noted by the reviewer, our interpretation of compartmentalized signaling is based on detecting total cellular cAMP by selectively modulating expression of compartment-specific PDEs rather than on direct visualization of localized cAMP gradients. Therefore, we cannot rule out that total cellular cAMP concentration may also influence

certain processes, such as parasite proliferation. To reflect this, we have modified the discussion to include this possibility (lines: 521-529).

2. Relevant to the above point, have the authors considered using PDEC (modified to localise to the flagellum) as an add back in to PDEB1/2 KO line? Since PDEC activity contributes dominantly to total cellular cAMP levels, this could establish if it is the level or the location of the cAMP that drives the observed phenotypes, or the specificity and localisation of the PDE isoform.

We appreciate the reviewer's insightful suggestion. We agree that a targeted relocation of PDE would be an interesting strategy to test whether the specific localization of PDE or total cAMP levels determines the observed phenotypes. However, changing the localization of the PDE will not affect the localization of the cAMP that drives the affected phenotypes, as these signals are generated by adenylate cyclases TcAC1 and TcAC2, modulated by TcCARP3, that we have previously described as dually localized at the CVC and the flagellar tip (references 27 and 28). Disruption of TcPDEC localization by deletion of its FYVE motif has been previously investigated (Ref 43) and the RVD phenotype of the mutant was similar to that observed in the presence of a specific PDEC inhibitor. In addition, flagellar localization signals remain unknown in *T. cruzi*, which limits our ability to relocate PDEC to the flagellum. For that reason, our working model is that compartment-specific PDEs prevents the diffusion of cAMP generated within distinct compartments, thereby maintaining spatially regulated signaling. Consistent with this idea, removal of compartment-specific PDE activity produces clearly distinct phenotypes across multiple stages of the parasite life cycle. Based on this model, we predict that relocating a catalytically active PDEC to the flagellum in the PDEB1/2-KO background will still preserve the FT compartment by preventing FT-cAMP diffusion, while disrupting the CVC compartment, therefore exhibiting the same phenotype as PDEC-KO parasites. However, PDEs could be regulated by compartment-specific modulators. Although it would be interesting to relocate PDEs, due to the technical limitations described above, generating a flagellar PDEC mutant in the PDEB1/2-KO background is beyond the scope of the present study.

3. Finally, do the authors consider that PDEB1 and 2 are fully functionally redundant or are there differences in their relative contribution?

In addition to their high sequence identity (82%), previous enzymatic characterization of these enzymes has shown that PDEB1 and PDEB2 exhibit a similar affinity for cAMP (Km 11.2 µM and 5.8–7.3 µM, respectively, reviewed in reference 55), and high substrate specificity. Consistent with this, we observed that PDEB1 and PDEB2 overexpression and addback mutants show similar phenotypes for cAMP content, cell growth, adhesion, metacyclogenesis, and osmoregulation, suggesting redundant functions within the flagellar compartment. We now mention this explicitly in the revised manuscript to clarify functional redundancy between PDEB1 and PDEB2 (lines: 290-293).

**Minor Issues:**

Fig. 1. PDEB1 and 2 are HA-tagged, and the rest of the PDEs are MYC-tagged. Even though all the proteins were localised to the expected region, could the authors confirm the tag does not affect localisation?

In this study, PDEB1 and PDEB2 were tagged with an HA epitope, whereas PDEA, PDEC, and PDED were tagged with c-Myc to either establish or confirm their localization. In addition, we confirmed the original localization of PDEB1, PDEB2, and PDEC using the Ty1 tag in the corresponding addback cell lines (Fig. S2). Importantly, the subcellular localization of PDEB1, PDEB2, and PDEC has also been previously reported using specific antibodies (references 43, 58, and 59), which we confirmed in the present study. Taken together, the consistent localization observed across multiple tagging strategies and previous studies supports the conclusion that the epitope tags used here do not alter the localization of these proteins.

Fig3.G-H. Could the authors show the "untrimmed" Western blots to demonstrate there no additional/unspecific bands. Also, it would be nice to see the image of the identical Western blot probed with an antibody against a protein with a constant expression level (i.e. a loading control). In the absence of this, please show the full image of the Ponceau-stained membrane. These images can be part of the Supplementary material.

Untrimmed western blots and a full image of the Ponceau-stained membrane have been provided as Supplementary material (Figure S5).

Fig3.I-J-K. Please Include the tagged cell lines in the bar charts (I- PDEC Myc; J-PDEB1 HA and PDEB2 HA). Also, please show the Western blot (with loading control) to accompany the bar charts; this would show the expression levels of PDEs in the tagged wt cell line vs the overexpressing cell lines.

We thank the reviewer for this suggestion. To clarify the identity of the cell lines used in these panels, we have added the tag information in the figure legend to clearly indicate which constructs contain HA or Ty1-tagged proteins. Indeed, there is no PDEC-3xc-Myc in Fig 3I as this endogenously tagged cell line was used for localization purposes only (Fig. 1D). The corresponding western blot analyses confirming expression of the tagged proteins are already presented in other figure panels; PDEB1/2 overexpression in Figure 1C, PDEC overexpression in Figure S2E, and the addback constructs in Figures 3G and 3H. To avoid duplication of western blot images, we refer to these existing panels rather than repeating them in Figure 3I–K. We have now clarified this in the figure legend and manuscript text.

Fig 4C. The growth rate measurement is represented in a strange format (Growth rate 1/day). Perhaps rather than growth per day these should be presented relative to wild type growth?

The growth rate charts have been reformatted as relative growth rate to facilitate the visualization and interpretation of this phenotype in Figures 4C and 4D, as suggested.

Fig 4F. In the text the enhanced level of metacyclic formation upon PDEB1/2 KO is described as a 'defect'. Perhaps better to describe it as a 'phenotype'? Also, on Fig 3J, the PDEB 1 and 2 ABs reduced the relative cAMP content compared to the control cell line, but on Fig4.F the ABs have the same ability to form metacyclics as the control cell line. Please explain.

We thank the reviewer for raising this concern. We agree that the term “phenotype” is appropriate in this context and have replaced the word (line: 249). Regarding the apparent discrepancy between the reduced total cAMP levels observed in the PDEB1 and PDEB2 addback lines (Fig. 3J) and the metacyclogenesis phenotype (Fig. 4F), our explanation is that increased levels of FT-cAMP such those observed in PDEB1/2 KO parasites stimulate metacyclogenesis by cAMP binding to unknown effectors in the flagellar compartment. However, the opposite effect does not necessarily occur. Indeed, we do not expect that a decrease in FT-cAMP compared to basal levels in control parasites would impair their ability to differentiate into metacyclic forms, as the synthesis of the second messenger under stress conditions will not be affected in the addback mutants. cAMP signaling relies on conformational changes of effectors upon cAMP binding. Therefore, cAMP levels lower than basal should not cause defects in *T. cruzi* life cycle. Indeed, PDEB1 and PDEB2 overexpression and addback cell lines did not show phenotypes in cell growth (Fig. 4B,D and Fig. S3A,B), osmoregulation (Fig. 5D,G), and adhesion (Fig. S3C). Another possible explanation is that parasites undergo metacyclogenesis only in response to threshold-dependent changes in cAMP signaling, where only larger perturbations produce detectable phenotypes.

In Fig 5 H and I (PDEB1 and 2 OE tracks) the violin plots suggest the potential for two subpopulations (high and low volume change or recovery). As it is at the population level no significant difference is observed with either OE line, but if there are two subpopulations (for example, some cells express the transgene better than others) a difference might become apparent?

The data points in the violin plots (Fig. 5H,I) correspond to 3 biological replicates from clonal populations of the four cell lines (T7/Cas9, PDEB1-OE, PDEB2-OE, and PDEC-OE). Importantly, the expression of PDEB1 and PDEB2 in overexpression cell lines was analyzed by both immunofluorescence and western blot analyses, where we confirmed the expression of these proteins along the flagellum (Fig 1C,D). Therefore, the data distribution in the violon plots reflects normal experimental variability within clonal populations, rather than biologically distinct subpopulations. Consistent with this, no significant differences were observed between PDEB1-OE or PDEB2-OE parasites compared to the control.

In Fig 7, PDEC KO generates reduced infectivity of the triatomine bug. Could this be due to the slower growth phenotype observed in Figure 4A?

We agree that the reduced hindgut colonization observed in TcPDEC-KO parasites could be influenced, at least in part, by their slower growth phenotype (Fig. 4A). However, we believe that the vector colonization phenotype of PDEC-KO could be a consequence of multiple factors. First of all, PDEB1/2-KO parasites had no growth defect (Fig. 4D), but showed significantly enhanced colonization in the insect vector, a phenotype that correlates with their increased *in vitro* differentiation into infective metacyclic trypomastigotes (Fig 4F). In contrast, TcPDEC-KO parasites exhibit a growth defect (Fig. 4C) but do not show an altered metacyclogenesis phenotype. Instead, these parasites display a marked defect in hyperosmotic stress tolerance, which is consistent with a reduced ability to adapt to the hindgut environment (Fig 7C, D). Importantly, in our vector colonization assay, insects were blood-fed with equal numbers of epimastigotes from the control, knockout, and addback cell lines to minimize the effect of differences in parasite abundance at the start of infection. Thus, while impaired response to hyperosmotic stress is likely the cause of the reduced vector colonization, we cannot rule out that slower parasite proliferation

may be also playing a role in this defect. We have included this explanation in the revised discussion of the manuscript (lines: 472-478).

Fig 7C. and line 316: "Interestingly, we observed that TcPDEC-KO parasites experienced a significantly higher shrinkage than control and AB cell lines upon hyperosmotic stress (Fig. 7C, D), being unable to recover their normal volume even after 15 min." According to Fig 7C, after 700sec (11.7 min), neither TcPDEC-KO nor AB cell lines can recover. Please correct this, and/or explain.

We thank the reviewer for pointing out this inconsistency. We agree that the original wording was inaccurate. The time course shown in Fig. 7C extends to approximately 18 minutes, corresponding to 15 minutes after induction of hyperosmotic stress. As shown in the figure, none of the parasite lines fully recovered their original volume during the assay. The phenotype we intended to highlight is the difference between TcPDEC-KO and TcPDEC-AB parasites during the interval between 500 and 600 seconds, which is the time window quantified in Fig. 7D. In this time range, TcPDEC-AB parasites show partial recovery relative to TcPDEC-KO parasites. We have revised the text (lines: 328-330) to accurately reflect the time window in which this difference was observed.

In the text, some statements are a little too strong. For example, it is stated PDEC is required for vector colonization. But the evidence is that it is *important* for efficiency but not *required* since there is still a level of colonisation, albeit reduced.

We thank the reviewer for this helpful suggestion. We agree that the term "required" overstates our findings. We have replaced "required" with "important" and have been updated accordingly (line 138, 341).

As mentioned earlier, the authors in the discussion highlight that their experiments allow them to selectively modulate the cAMP levels in different cellular compartments. This likely but is not demonstrated, only inferred. I suggest to be a little more cautious.

We thank the reviewer for this helpful suggestion and agree that our conclusions regarding compartment-specific modulation of cAMP signaling are based on the modulated expression of phosphodiesterases localized to distinct subcellular compartments, rather than direct measurement of localized cAMP concentrations. To address this concern, we have revised the relevant statements throughout the manuscript, as highlighted in the marked-up version.

**Reviewer #2**

**Summary:** In this manuscript, Ahmed et al. investigate the role of phosphodiesterase-defined cAMP signaling compartments in *Trypanosoma cruzi* and propose the existence of two functionally distinct cAMP microdomains localized to the flagellum (FT-cAMP) and the contractile vacuole complex (CVC-cAMP). Using genetic ablation and overexpression of PDEB1/2 and PDEC, the authors link these compartments to specific biological processes, including metacyclogenesis, host cell invasion, intracellular replication, osmoregulation, and vector colonization.

The study is technically solid and addresses an important question in parasite signal transduction with clear relevance for infection biology and transmission. The genetic toolkit is well applied, and the phenotypic analyses are comprehensive and biologically meaningful. In particular, the identification of a flagellar signaling module controlling differentiation and infectivity represents a significant advance.

However, the central claim of compartmentalized "cAMP microdomains" is supported only indirectly. The authors measure only total cellular cAMP levels and rely primarily on PDE localization and phenotypic segregation to infer the existence of spatially restricted cAMP pools. No spatially resolved cAMP measurements or downstream effector localization are provided. Thus, while the data strongly support functionally distinct PDE-defined signaling compartments, they fall short of demonstrating bona fide cAMP microdomains as defined in the classical literature.
Overall, the manuscript is suitable for publication after major revision, provided that the authors temper their claims and address the conceptual and interpretative limitations outlined below.

We thank the reviewer for their thoughtful and constructive evaluation of our manuscript, and we appreciate their recognition of the significance of our work. We also appreciate the reviewer's critical perspective regarding the interpretation of cAMP signaling microdomains. To address this concern, we have revised the manuscript to moderate our claims and better align the conceptual framework with the experimental evidence.

**Major Issues:**

1. Evidence for cAMP microdomains is indirect:

The manuscript's main conceptual claim is the existence of compartmentalized cAMP microdomains at the flagellar tip and the contractile vacuole complex. However, only total cellular cAMP levels were measured. No spatially resolved cAMP signals or dynamics were demonstrated (e.g. via FRET/EPAC sensors, subcellular fractionation, or localized reporters).

At present, the data demonstrate PDE-specific functional compartments rather than true cAMP microdomains. The authors should either: (a) temper their language throughout the manuscript (e.g., "putative microdomains" or "PDE-defined signaling compartments"), and/or (b) provide additional experimental evidence that directly supports localized cAMP pools.

This point affects the Abstract, Results, and Discussion and should be addressed consistently.

We agree with the reviewer that our data provide indirect rather than direct evidence for spatially restricted cAMP signaling. As mentioned in the original manuscript, the direct measurement of compartment-specific cAMP dynamics requires approaches such as FRET-based cAMP reporters. To address the reviewer's concern, we have revised the manuscript to moderate the terminology used throughout the text. Specifically, the term *"cAMP microdomains"* has been replaced with *"PDE-defined cAMP compartments"* or similar phrasing where appropriate. Because PDEB1, PDEB2, and PDEC have been previously shown to be cAMP-specific PDEs, we consider appropriate referring to these compartments as *PDE-defined cAMP compartments*. In addition, the *Abstract*, *Introduction*, *Results*, and *Discussion* sections have been revised to clarify that our

conclusions are based on functional perturbation of compartment-specific PDEs, rather than direct visualization of localized cAMP pools.

2. Localization of PDEs does not establish compartmentalized cAMP signaling per se: The microscopy data convincingly show that PDEB1/2 localize to the flagellum and PDEC to the CVC. However, these images show only protein localization and do not reveal any structural or organizational features that would explain how functional signaling domains are formed or insulated from the cytosol.

No evidence is provided for either local cAMP gradients, diffusion barriers, co-localization with downstream effectors (e.g., PKA or CARP proteins), or architectural scaffolding. Thus, the jump from enzyme localization to the existence of functional microdomains remains inferential. This limitation should be explicitly acknowledged and discussed. I do not suggest additional experiments, as they would be highly demanding (but I would be curious to them in future).

We agree with the reviewer that enzyme localization together with functional segregation, does not directly demonstrate the existence of insulated cAMP signaling microdomains. This limitation was acknowledged in the discussion of the original manuscript. In the revised manuscript, we have moderated our interpretation accordingly. Specifically, we now state in the *Results* (lines: 231-234) and *Discussion* (lines 521–529) that our findings are consistent with spatially restricted cAMP signaling, but do not directly demonstrate local cAMP gradients or the structural basis of signaling insulation. We have also revised the text throughout the manuscript to avoid wording that implies direct proof of microdomain architecture. We replaced the term "cAMP microdomains" with "PDE-defined cAMP compartments" where appropriate.

Importantly, our interpretation is not based solely on PDE localization. Previous work from our laboratory showed that the adenylate cyclases TcAC1 and TcAC2, which synthesize cAMP, as well as the cAMP response protein TcCARP3, exhibit dual localization to both the flagellar tip and the contractile vacuole complex. Building on those findings, the present study selectively perturbs compartment-associated phosphodiesterases to functionally dissect the roles of these compartments in regulating cAMP-dependent processes. Thus, our conclusions are based on structural evidence (references 27, 28, 58, 59, 60), and new functional evidence derived from compartment-specific perturbation of PDE activity, which reveals distinct biological outputs associated with these compartments, rather than on direct visualization of compartmentalized cAMP pools.

Finally, we have revised the manuscript throughout to emphasize that direct measurement of compartment-specific cAMP dynamics, as well as identification of the mechanisms underlying signaling insulation, such as diffusion barriers, co-localized downstream effectors, or architectural scaffolds, remain important directions for future study for complete characterization of signaling microdomains (lines: 540-545).

3. Causality between localization and function is not directly tested:

While PDEC contains a FYVE domain that plausibly mediates CVC targeting, no experiments test whether localization itself is required for the observed phenotypes (e.g., mislocalized PDE mutants). For PDEB1/2, no defined targeting motif is known or functionally examined.

A key test of the microdomain model would be to show that enzymatic activity at a different location produces different phenotypes. In the absence of such experiments, conclusions about location-dependent signaling remain correlational. This conceptual limitation should be addressed in the Discussion. Also, a FYVE domain mutant would probably be very informative.

We acknowledge the reviewer's observation and agree that experiments testing whether enzyme localization is required for the observed phenotypes by targeting the domain would provide stronger evidence for localization-dependent signaling. However, previous work has already addressed the role of the FYVE domain in TcPDEC localization (reference 43). In this work the authors demonstrated that parasites expressing a truncated PDEC lacking the FYVE domain failed to localize to the CVC and exhibited markedly reduced phosphodiesterase activity and an osmoregulatory defect, supporting the importance of this domain for both correct localization and enzymatic function. Because these experiments have already established the role of the FYVE domain in PDEC targeting and function, we did not revisit this approach in the present study. Flagellar localization signals remain unexplored in *T. cruzi*. Defining them was outside the scope of the present study. We have addressed this limitation in the new version of the manuscript (lines: 531-538).

4. Interpretation of vector colonization phenotype remains partly speculative.

The link between impaired hyperosmotic stress tolerance in TcPDEC-KO parasites and reduced hindgut colonization in *Rhodnius prolixus* is plausible and interesting but remains inferential. No direct in vivo measurement of osmotic stress or rescue experiment in the vector is provided. I know this is very challenging.

The authors should tone down causal language and frame this connection more cautiously as a correlation supported by physiological assays.

We appreciate the reviewer's observation and agree that the connection between impaired response to hyperosmotic stress in TcPDEC-KO parasites and reduced hindgut colonization is inferential. However, the hyperosmotic environment of the vector's hindgut has been previously reported (reference 66). in addition, the PDEC-AB cell line was included as control and partially rescued both phenotypes (defect in hindgut colonization and hyperosmotic stress), suggesting a specific role for PDEC in both processes. As acknowledged by the reviewer, these experiments are very challenging, and our intention was to provide a possible explanation to an unexpected result. We have revised the discussion to further clarify our interpretation of these results (lines: 472-478).

Strength of conclusions should be moderated
Throughout the manuscript, the authors use relatively strong language such as "unequivocally demonstrate" and "microdomains drive signal specificity." Given the indirect nature of the evidence for localized cAMP signaling, these statements should be moderated to reflect the current level of experimental support. This would be no means lower the impact of the publication-

We have carefully revised the manuscript to moderate statements. Phrases such as "unequivocally demonstrate" have been replaced with more cautious language that better reflects the current experimental evidence.

**Minor Issues:**

1. Use of the term "microdomain" in the Abstract

The term "cAMP microdomains" is used before any spatial definition is provided. Consider using "putative microdomains" or "signaling compartments" in the abstract.

We agree and have revised the Abstract to replace the term "cAMP microdomains" with "PDE-defined cAMP signaling compartments".

2. Clarification of "flagellar tip (FT)" versus entire flagellum

lines 130–135: PDEB1/2 appear distributed along the flagellum, yet the text refers to a "flagellar tip" microdomain. Please clarify whether this refers to the distal tip or the whole flagellum.

We thank the reviewer for raising this important point. PDEB1 and PDEB2 are indeed distributed along the length of the flagellum, as shown by our localization data. However, the cAMP signaling compartment we refer to as the flagellar tip (FT) corresponds to the distal region of the flagellum, where adenylyl cyclases AC1 and AC2, as well as the cAMP response protein CARP3, have been shown to localize and form a functional cAMP compartment (references 27 and 28). In this context, PDEB1 and PDEB2, localized along the flagellum, would prevent the diffusion of cAMP generated at the flagellar tip, thereby contributing to the spatial regulation of cAMP signaling associated with this compartment. To avoid confusion, we have clarified this point (lines 128-130) and now explicitly state that PDEB1 and PDEB2 localization extends along the flagellum to regulate the FT-cAMP compartment.

3. Lack of quantitative localization analysis
lines 167–190: The microscopy data are qualitative. line-scan profiles or enrichment ratios (flagellum vs cytosol; CVC vs cytosol) would strengthen the localization claims.

We agree that quantitative localization analyses, such as line-scan profiles or enrichment measurements, can provide additional support for subcellular localization patterns. However, the localization of PDEB1/2 and TcPDEC has been previously reported by IFA and cryo-electron microscopy using specific antibodies (References 43, 58, and 59). In the present study, our goal was to confirm the previously reported localization of PDEB1, PDEB2, and TcPDEC for functional characterization of CVC and FT cAMP compartments. This validation allowed us to use these PDEs as compartment-specific functional markers to selectively modulate cAMP signaling in the flagellar tip and contractile vacuole complex. However, we acknowledge that further microscopy analyses should be performed to confirm the localization of PDEA and PDED, but these PDEs were not the main focus of the present study, and this will be the subject of future research in our lab.

4. Overexpression versus endogenous tagging should be clearly stated

lines 168–176; Fig. 1 and Fig. 2 legends: PDEB1/2 localization relies on overexpression constructs, whereas other PDEs are endogenously tagged. Please explicitly indicate this in the figure legends and briefly discuss potential artifacts.

We have updated the details of the constructs in the figure legends as suggested. We did mention in the manuscript that PDEA, PDEC, and PDED were endogenously tagged while PDEB1 and PDEB2 were overexpressed (lines 172–177). Our observations here are consistent with previous reports and we did not observe any artifacts in the localization analyses.

5. Justification of rescue strategy
lines 284–287: The use of PDEB2-AB as rescue control (instead of PDEB1-AB or dual addback) requires further justification. Please clarify why a combined or expression-matched rescue was not used.

Several considerations guided our choice of PDEB2-AB as the rescue control in the mammalian cell invasion and intracellular replication assays. First, our data suggest that PDEB1 and PDEB2 have largely redundant functions. They are tandemly organized genes that share high sequence similarity and comparable biochemical properties, and we did not observe clearly distinguishable phenotypes between PDEB1 and PDEB2 overexpression or addback cell lines in the assays performed. Most likely, PDEB1 and PDEB2 are duplicated genes with redundant roles in *T. cruzi*. Thus, adding back either enzyme was expected to provide a valid functional rescue of the PDEB1/2-KO invasion phenotype. The invasion and intracellular replication assays are extremely time consuming and involved the manipulation of the infective stage of the parasite. So, we randomly chose one of the addback cell lines as a rescue control. The successful rescue obtained with PDEB2-AB therefore supports the conclusion that the phenotype is specifically linked to loss of PDEB-associated cAMP regulation. A dual addback line was not pursued because of technical limitations related to selectable markers in *T. cruzi*. Generation of a dual rescue line in the already modified background would have required an additional selectable marker beyond those available in our system. We have now clarified this rationale in the revised *Results* section (lines: 290-293) and in *Materials and Methods* (lines: 634-636).

6. Limitations of total cAMP measurements
lines 217–225: Only total cellular cAMP is measured. This limitation should be acknowledged already in the Results section, not only in the Discussion.

We agree that this limitation should be acknowledged earlier in the manuscript. We have therefore added a clarifying statement in the *Results* section (lines: 231-234) indicating that the cAMP measurements presented reflect total cellular cAMP content rather than determining compartment-specific cAMP concentrations. The broader implications of this limitation for the interpretation of compartmentalized cAMP signaling are discussed further in the Discussion section.

7. Vector colonization assay lacks parasite burden quantification

lines 299–305: Please clarify whether parasite load per hindgut was quantified or whether only presence/absence was scored.

In the vector assays, colonization was evaluated by scoring the presence or absence of metacyclic trypomastigotes in the hindgut after dissection. In these assays, parasite load per insect was not

quantified due to the low parasite density observed in the hindguts. We have revised the result sections for clarification (lines: 309-312).

8. Speculative interpretation of osmotic stress effects

lines 312–320: The proposed causal link between hyperosmotic stress sensitivity and vector colonization could be phrased more cautiously.

We have been more cautious now with the interpretation of our results. In the revised manuscript, we now mention that the observed defect in response to hyperosmotic stress may contribute to the reduced vector colonization phenotype in addition to the growth defect observed in TcPDEC-KO parasites (lines: 472-478).

9. TcPDEC absence in metacyclics

lines 187–190: Please clarify whether the absence of detectable TcPDEC in metacyclic trypomastigotes reflects downregulation or technical detection limits.

In our localization experiments, TcPDEC was not detected in metacyclic trypomastigotes by IFA. Transcriptomic analyses across the main developmental stages of *T. cruzi* (PMID: 19664227) report PDEC transcripts in metacyclic trypomastigotes, suggesting that the lack of signal in our IFAs is due to low protein expression in this developmental stage, that is below the sensitivity threshold of standard epifluorescence microscopy. Another possibility is that PDE expression is post-transcriptionally regulated in metacyclic trypomastigotes. Further microscopy analyses by super-resolution, expansion microscopy, or cryoimmunoelectron microscopy should be performed to validate this result. We have now revised the *Results* section to state that TcPDEC was not detectable in metacyclic trypomastigotes in our assay (lines: 191-195).

10. TcPDEA and TcPDED not functionally explored

lines 179–183; Discussion 506–507:

Please briefly justify why these PDEs were not included in functional analyses.

In this study, our goal was functional characterization of the two previously proposed cAMP signaling compartments associated with the flagellar tip and the contractile vacuole complex (CVC), where our previous work identified adenylate cyclases TcAC1/TcAC2 and the cAMP response protein TcCARP3, supporting the existence of active cAMP signaling in these regions. Because PDEB1/2 and TcPDEC localize specifically to these compartments, they provided the most appropriate molecular tools to selectively perturb FT- and CVC-associated cAMP signaling, respectively. In contrast, TcPDEA and TcPDED showed distinct localization patterns outside these compartments in our analyses, and hence they were not further investigated in the present study. We agree, however, that these PDEs are of considerable interest, and we now mentioned in the Discussion (lines: 538-541) that future characterization of TcPDEA and TcPDED may reveal additional cAMP-regulated compartments or functions in *T. cruzi*.

11. Figure legends should include imaging parameters

Figures 1D and 2A–C: Please provide scale bars, magnification, and acquisition parameters.

Done.

12. Statistical reporting

Figures 4–7: Please indicate exact n values, number of biological replicates, and statistical tests in all figure legnends.

Done.

13. Terminology consistency Throughout Results and Discussion: Standardize usage of "FT-cAMP," "flagellar microdomain," "flagellar tip," and "flagellum."

We no longer use the term "flagellar microdomain" in the revised manuscript. We are referring to the FT compartment as FT-cAMP. However, FT-cAMP (the compartment), flagellar tip (the distal domain of the flagellum), and flagellum (cellular structure) are three conceptually different terms that we continue using when suitable in the manuscript.

**Reviewer #3**

**Summary:** This manuscript by Ahmed and colleagues presents a significant advance in our understanding of cAMP signaling in *Trypanosoma cruzi*. While the importance of this pathway for environmental sensing and stress response in trypanosomatids is well-established, the mechanisms underlying signal specificity have remained largely elusive. Here, the authors provide compelling experimental evidence to functionally validate the existence of two distinct and independent cAMP microdomains in the parasite: one at the flagellar tip (FT-cAMP) and another at the contractile vacuole complex (CVC-cAMP). By elegantly knockout and modulating the expression of compartment-specific phosphodiesterases (PDEB1/B2 and PDEC), they demonstrate a clear functional segregation. Their findings robustly link the FT-cAMP microdomain to critical steps in the mammalian host infection, such as cell adhesion, metacyclogenesis, and intracellular replication. Conversely, they show that the CVC-cAMP microdomain is involved in osmoregulation and modulate proliferation in the insect stage. The work is methodologically sound, the data strongly support the conclusions, and it provides the first robust evidence for spatially regulated cAMP signaling dictating specific cellular responses throughout the complex life cycle of *T. cruzi*. However, some minor points need to be clarified and corrected in the text to further strengthen the clarity of the manuscript

We thank the reviewer for their positive evaluation of our manuscript and for recognizing the significance and overall quality of our work.

**Minor Issues:**

It was unclear to me whether the results represent technical triplicates from three different clones (for each transfection) or biological triplicates from a single clone. Please clarify this clearly in the methodology section and in the figure legends.

We thank the reviewer for pointing out this lack of clarity. The reported values (n = 3) represent biological replicates performed using clonal populations, rather than technical replicates or multiple clones. We now mentioned this in the "Statistical analyses" of the Methods section (lines: 799-800) and in the figure legends to explicitly state that the experiments were performed using three biological replicates from the same clone.

lines 234-236: The description of the results should accurately reflect the statistical data presented in Fig. 4D. Although the growth rate decreased compared to the Cas9/T7 control, the AB lines failed to rescue the phenotype when compared to the KO lines, as clearly observed in Fig. 4C.

The growth data shown in Figures 4C and 4D, indicates that the addback cell lines restored the phenotype during the exponential phase of growth (days 2-7), with no significant differences observed related to control cells.

Figure 4: Please ensure that graphs depicting the same type of experiment use consistent axis scales for proper comparison.

Done when suitable, depending on the specific chart.

Figure 4F: Please include the statistical analysis comparing PDEB1/2-KO vs. PDEB1-AB and PDEB2-AB.

Done.

lines 257-259: The statement "Together, these results confirm that adhesion is a prerequisite for metacyclogenesis and that FT-cAMP signals, but not CVC-cAMP, mediate both processes in *T. cruzi*" may be too strong. Based on the data presented, it would be more appropriate to suggest that adhesion correlates with metacyclogenesis, rather than using the term "prerequisite," which implies a stricter causal relationship.

We thank the reviewer for this helpful suggestion and agree that the term "prerequisite" was too strong in this context. Our data support an association between enhanced adhesion and increased metacyclogenesis *in vitro* in the TcPDEB1/2-KO line. We have therefore revised the *Results* section (lines: 262-264) to state that adhesion correlates with metacyclogenesis under our experimental conditions, rather than describing it as a prerequisite.
