# Supplementary material for: Cyclic AMP compartmentalization drives signal specificity to control vector colonization and mammalian host infection by American trypanosomes
Source: PLoS Pathog. 2026 Jun 29;22(6):e1013784. doi: 10.1371/journal.ppat.1013784 (PMC13340794; doi:10.1371/journal.ppat.1013784)
Supplement: Peer review history

# Peer review: Decision Letter 0

**DOI:** 10.1371/journal.ppat.1013784.r001

**Authors:** Jeffrey Dvorin, Tim Nicolai Siegel

5 Mar 2026

-->PPATHOGENS-D-25-03089

Cyclic AMP compartmentalization drives signal specificity to control vector colonization and mammalian host infection by American trypanosomes

PLOS Pathogens

Dear Dr. Lander,

Thank you for submitting your manuscript to PLOS Pathogens. After careful consideration, we feel that it has merit but does not fully meet PLOS Pathogens's publication criteria as it currently stands. Therefore, we invite you to submit a revised version of the manuscript that addresses the points raised during the review process.

Please submit your revised manuscript by May 04 2026 11:59PM. If you will need more time than this to complete your revisions, please reply to this message or contact the journal office at plospathogens@plos.org. When you're ready to submit your revision, log on to https://www.editorialmanager.com/ppathogens/ and select the 'Submissions Needing Revision' folder to locate your manuscript file.

Please include the following items when submitting your revised manuscript:

* A letter that responds to each point raised by the editor and reviewer(s). You should upload this letter as a separate file labeled 'Response to Reviewers'. This file does not need to include responses to any formatting updates and technical items listed in the 'Journal Requirements' section below.

* A marked-up copy of your manuscript that highlights changes made to the original version. You should upload this as a separate file labeled 'Revised Manuscript with Track Changes'.

* An unmarked version of your revised paper without tracked changes. You should upload this as a separate file labeled 'Manuscript'.

If you would like to make changes to your financial disclosure, competing interests statement, or data availability statement, please make these updates within the submission form at the time of resubmission. Guidelines for resubmitting your figure files are available below the reviewer comments at the end of this letter.

We look forward to receiving your revised manuscript.

Kind regards,

Tim Nicolai Siegel, Ph.D

Academic Editor

PLOS Pathogens

Jeffrey Dvorin

Section Editor

PLOS Pathogens

Sumita Bhaduri-McIntosh

Editor-in-Chief

PLOS Pathogens

orcid.org/0000-0003-2946-9497

Michael Malim

Editor-in-Chief

PLOS Pathogens

orcid.org/0000-0002-7699-2064

**Journal Requirements:**

1) We do not publish any copyright or trademark symbols that usually accompany proprietary names, eg ©,  ®, or TM  (e.g. next to drug or reagent names). Therefore please remove all instances of trademark/copyright symbols throughout the text, including:

- ® on pages: 24, and 26

- TM on page: 24.

2) Some material included in your submission may be copyrighted. According to PLOSu2019s copyright policy, authors who use figures or other material (e.g., graphics, clipart, maps) from another author or copyright holder must demonstrate or obtain permission to publish this material under the Creative Commons Attribution 4.0 International (CC BY 4.0) License used by PLOS journals. Please closely review the details of PLOSu2019s copyright requirements here: PLOS Licenses and Copyright. If you need to request permissions from a copyright holder, you may use PLOS's Copyright Content Permission form.

Please respond directly to this email and provide any known details concerning your material's license terms and permissions required for reuse, even if you have not yet obtained copyright permissions or are unsure of your material's copyright compatibility. Once you have responded and addressed all other outstanding technical requirements, you may resubmit your manuscript within Editorial Manager.

Potential Copyright Issues:

i) Figure 8. Please confirm whether you drew the images / clip-art within the figure panels by hand. If you did not draw the images, please provide (a) a link to the source of the images or icons and their license / terms of use; or (b) written permission from the copyright holder to publish the images or icons under our CC BY 4.0 license. Alternatively, you may replace the images with open source alternatives. See these open source resources you may use to replace images / clip-art:

- https://commons.wikimedia.org

- https://openclipart.org/.

3) We note that your Data Availability Statement is currently as follows: "All relevant data are in the manuscript and its Supporting information files.". Please confirm at this time whether or not your submission contains all raw data required to replicate the results of your study. Authors must share the “minimal data set” for their submission. PLOS defines the minimal data set to consist of the data required to replicate all study findings reported in the article, as well as related metadata and methods (https://journals.plos.org/plosone/s/data-availability#loc-minimal-data-set-definition).

For example, authors should submit the following data:

1) The values behind the means, standard deviations and other measures reported;

2) The values used to build graphs;

3) The points extracted from images for analysis..

Authors do not need to submit their entire data set if only a portion of the data was used in the reported study.

If your submission does not contain these data, please either upload them as Supporting Information files or deposit them to a stable, public repository and provide us with the relevant URLs, DOIs, or accession numbers. For a list of recommended repositories, please see https://journals.plos.org/plosone/s/recommended-repositories.

If there are ethical or legal restrictions on sharing a de-identified data set, please explain them in detail (e.g., data contain potentially sensitive information, data are owned by a third-party organization, etc.) and who has imposed them (e.g., an ethics committee). Please also provide contact information for a data access committee, ethics committee, or other institutional body to which data requests may be sent. If data are owned by a third party, please indicate how others may request data access.

4) Please amend your detailed Financial Disclosure statement. This is published with the article. It must therefore be completed in full sentences and contain the exact wording you wish to be published.

1) Since the funders had no role in your study, please state: "The funders had no role in study design, data collection and analysis, decision to publish, or preparation of the manuscript.".

Note: If the reviewer comments include a recommendation to cite specific previously published works, please review and evaluate these publications to determine whether they are relevant and should be cited. There is no requirement to cite these works unless the editor has indicated otherwise.

**Reviewers' Comments:**

Reviewer's Responses to Questions

**Part I - Summary**

Please use this section to discuss strengths/weaknesses of study, novelty/significance, general execution and scholarship.

Reviewer #1: This manuscript investigates the contribution of localised cAMP levels to T. cruzi growth, development, resistance to osmotic stress and vector infection by perturbing the levels of PDEs that have specific cellular location in the parasite. The experiments are well described and good quality, with good use of knockout, addback and overexpression approaches to confirm or explore in more detail observed phenotypes. We only have a few comments that the authors could consider.

Reviewer #2: In this manuscript, Ahmed et al. investigate the role of phosphodiesterase-defined cAMP signaling compartments in Trypanosoma cruzi and propose the existence of two functionally distinct cAMP microdomains localized to the flagellum (FT-cAMP) and the contractile vacuole complex (CVC-cAMP). Using genetic ablation and overexpression of PDEB1/2 and PDEC, the authors link these compartments to specific biological processes, including metacyclogenesis, host cell invasion, intracellular replication, osmoregulation, and vector colonization.

The study is technically solid and addresses an important question in parasite signal transduction with clear relevance for infection biology and transmission. The genetic toolkit is well applied, and the phenotypic analyses are comprehensive and biologically meaningful. In particular, the identification of a flagellar signaling module controlling differentiation and infectivity represents a significant advance.

However, the central claim of compartmentalized “cAMP microdomains” is supported only indirectly. The authors measure only total cellular cAMP levels and rely primarily on PDE localization and phenotypic segregation to infer the existence of spatially restricted cAMP pools. No spatially resolved cAMP measurements or downstream effector localization are provided. Thus, while the data strongly support functionally distinct PDE-defined signaling compartments, they fall short of demonstrating bona fide cAMP microdomains as defined in the classical literature.

Overall, the manuscript is suitable for publication after major revision, provided that the authors temper their claims and address the conceptual and interpretative limitations outlined below.

Reviewer #3: This manuscript by Ahmed and colleagues presents a significant advance in our understanding of cAMP signaling in Trypanosoma cruzi. While the importance of this pathway for environmental sensing and stress response in trypanosomatids is well-established, the mechanisms underlying signal specificity have remained largely elusive. Here, the authors provide compelling experimental evidence to functionally validate the existence of two distinct and independent cAMP microdomains in the parasite: one at the flagellar tip (FT-cAMP) and another at the contractile vacuole complex (CVC-cAMP). By elegantly knockout and modulating the expression of compartment-specific phosphodiesterases (PDEB1/B2 and PDEC), they demonstrate a clear functional segregation. Their findings robustly link the FT-cAMP microdomain to critical steps in the mammalian host infection, such as cell adhesion, metacyclogenesis, and intracellular replication. Conversely, they show that the CVC-cAMP microdomain is involved in osmoregulation and modulate proliferation in the insect stage. The work is methodologically sound, the data strongly support the conclusions, and it provides the first robust evidence for spatially regulated cAMP signaling dictating specific cellular responses throughout the complex life cycle of T. cruzi. However, some minor points need to be clarified and corrected in the text to further strengthen the clarity of the manuscript

**********

**Part II – Major Issues: Key Experiments Required for Acceptance**

Please use this section to detail the key new experiments or modifications of existing experiments that should be absolutely required to validate study conclusions.

Generally, there should be no more than 3 such required experiments or major modifications for a "Major Revision" recommendation. If more than 3 experiments are necessary to validate the study conclusions, then you are encouraged to recommend "Reject".

Reviewer #1: Mainly, as highlighted by the authors in their discussion, the cAMP measurements are whole cell assays, while the PDE KO effects are expected to be local. Although local cAMP concentrations (dependent on local PDE levels) likely explain the phenotypes, it is also possible that there is a calibrated response at the whole cell level, with observed and different phenotypes dependent on the absolute cellular cAMP level. As an example, a growth effect is attributed to the CV PDEC acting locally on cAMP levels, but it could also be a response to overall cellular cAMP levels, which is dominantly controlled by PDE1C. As the authors suggest, local cAMP concentrations could be directly assayed by FRET based approaches (or other reporters) but I don’t think this is necessary in this manuscript. However, the possibility that whole cell cAMP levels (with different phenotypes manifest at different absolute levels) is worth discussing in the manuscript.

Relevant to the above point, have the authors considered using PDEC (modified to localise to the flagellum) as an add back in to PDEB1/2 KO line? Since PDEC activity contributes dominantly to total cellular cAMP levels, this could establish if it is the level or the location of the cAMP that drives the observed phenotypes, or the specificity and localisation of the PDE isoform.

Finally, do the authors consider that PDEB1 and 2 are fully functionally redundant or are there differences in their relative contribution?

Reviewer #2: 1. Evidence for cAMP microdomains is indirect:

Tthe manuscript’s main conceptual claim is the existence of compartmentalized cAMP microdomains at the flagellar tip and the contractile vacuole complex. However, only total cellular cAMP levels were measured. No spatially resolved cAMP signals or dynamics were demonstrated (e.g. via FRET/EPAC sensors, subcellular fractionation, or localized reporters).

At present, the data demonstrate PDE-specific functional compartments rather than true cAMP microdomains. The authors should either: (a) temper their language throughout the manuscript (e.g., “putative microdomains” or “PDE-defined signaling compartments”), and/or (b) provide additional experimental evidence that directly supports localized cAMP pools.

This point affects the Abstract, Results, and Discussion and should be addressed consistently.

2. Localization of PDEs does not establish compartmentalized cAMP signaling per se:

The microscopy data convincingly show that PDEB1/2 localize to the flagellum and PDEC to the CVC. However, these images show only protein localization and do not reveal any structural or organizational features that would explain how functional signaling domains are formed or insulated from the cytosol.

No evidence is provided for either local cAMP gradients, diffusion barriers, co-localization with downstream effectors (e.g. PKA or CARP proteins), or architectural scaffolding. Thus, the jump from enzyme localization to the existence of functional microdomains remains inferential. This limitation should be explicitly acknolwedged and discussed. I do not suggest additional experiments, as they would be highly demanding (but I would be curious to them in future).

3. Causality between localization and function is not directly tested:

While PDEC contains a FYVE domain that plausibly mediates CVC targeting, no exeperiments test whether localization itself is required for the observed phenotypes (e.g., mislocalized PDE mutants). For PDEB1/2, no defined targeting motif is known or functionally examined.

A key test of the microdomain model would be to show that enzymatic activity at a different location produces different phenotypes. In the absence of such experiments, conclusions about location-dependent signaling remain correlational. This conceptual limitation should be addressed in the Discussion. Also, a FYVE domain mutant would probably be very informative.

4. Interpretation of vector colonization phenotype remains partly speculative

The link between impaired hyperosmotic stress toelrance in TcPDEC-KO parasites and reduced hindgut colonization in Rhodnius prolixus is plausible and interesting, but remains inferential. No direct in vivo measurement of osmotic stress or rescue experiment in the vector is provided. In know this is very challenging.

The authors should tone down causal language and frame this connection more cautiously as a correlation supported by physiological assays.

5. Strength of conclusions should be moderated

Throughout the manuscript, the authors use relatively strong lanuaage such as “unequivocally demonstrate” and “microdomains drive signal specificity.” Given the indirect nature of the evidence for localized cAMP signaling, these statements should be moderated to reflect the current level of experimental support. This would be no means lower the impact of the publication-

Reviewer #3: (No Response)

**********

**Part III – Minor Issues: Editorial and Data Presentation Modifications**

Please use this section for editorial suggestions as well as relatively minor modifications of existing data that would enhance clarity.

Reviewer #1: Fig1.

PDEB1 and 2 are HA-tagged, and the rest of the PDEs are MYC-tagged.

Even though all the proteins were localised to the expected region, could the authors confirm the tag does not affect localisation?

Fig3.G-H.

Could the authors show the “untrimmed” Western blots to demonstrate there no additional/unspecific bands. Also, it would be nice to see the image of the identical Western blot probed with an antibody against a protein with a constant expression level (i.e. a loading control). In the absence of this, please show the full image of the Ponceau-stained membrane. These images can be part of the Supplementary material.

Fig3.I-J-K

Please Include the tagged cell lines in the bar charts (I- PDEC Myc; J-PDEB1 HA and PDEB2 HA). Also, please show the Western blot (with loading control) to accompany the bar charts; this would show the expression levels of PDEs in the tagged wt cell line vs the overexpressing cell lines.

Fig 4C The growth rate measurement is represented in a strange format (Growth rate 1/day). Perhaps rather than growth per day these should be presented relative to wild type growth?

Fig4.F

In the text the enhanced level of metacyclc formation upon PDEB1/2 KO is described as a ‘defect’. Perhaps better to describe it as a ‘phenotype’? Also, on Fig 3J, the PDEB 1 and 2 ABs reduced the relative cAMP content compared to the control cell line, but on Fig4.F the ABs have the same ability to form metacyclics as the control cell line. Please explain.

In Fig 5 H and I (PDEB1 and 2 OE tracks) the violin plots suggest the potential for two subpopulations (high and low volume change or recovery). As it is at the population level no significant difference is observed with either OE line, but if there are two subpopulations (for example, some cells express the transgene better than others) a difference might become apparent?

In Fig 7, PDEC KO generates reduced infectivity of the triatomine bug. Could this be due to the slower growth phenotype observed in Figure 4A?

Fig7C. and line 316:

“Interestingly, we observed that TcPDEC-KO parasites experienced a significantly higher shrinkage than control and AB cell lines upon hyperosmotic stress (Fig. 7C, D), being unable to recover their normal volume even after 15 min.”

According to Fig 7C, after 700sec (11.7 min), neither TcPDEC-KO nor AB cell lines can recover. Please correct this, and/or explain.

In the text, some statements are a little too strong. For example, it is stated PDEC is required for vector colonization. But the evidence is that it is *important* for efficiency but not *required* since there is still a level of colonisation, albeit reduced.

As mentioned earlier, the authors in the discussion highlight that their experiments allow them to selectively modulate the cAMP levels in different cellular compartments. This likely but is not demonstrated, only inferred. I suggest to be a little more cautious.

Reviewer #2: 1. Use of the term “microdomain” in the Abstract

The term “cAMP microdomains” is used before any spatial definition is provided. Consider using “putative microdomains” or “signaling compartments” in the abstract.

2. Clarification of “flagellar tip (FT)” versus entire flagellum

Lines 130–135:

PDEB1/2 appear distributed along the flagellum, yet the text refers to a “flagellar tip” microdomain. Please clarify whether this refers to the distal tip or the whole flagellum.

3. Lack of quantitative localization analysis

Lines 167–190:

The microscopy data are qualitative. Line-scan profiles or enrichment ratios (flagellum vs cytosol; CVC vs cytosol) would strengthen the localization claims.

4. Overexpression versus endogenous tagging should be clearly stated

Lines 168–176; Fig. 1 and Fig. 2 legends:

PDEB1/2 localization relies on overexpression constructs, whereas other PDEs are endogenously tagged. Please explicitly indicate this in figure legends and briefly discuss potential artifacts.

5. Justification of rescue strategy

Lines 284–287:

The use of PDEB2-AB as rescue control (instead of PDEB1-AB or dual addback) requires further justification. Please clarify why a combined or expression-matched rescue was not used.

6. Limitations of total cAMP measurements

Lines 217–225:

Only total cellular cAMP is measured. This limitation should be acknowledged already in the Results section, not only in the Discussion.

7. Vector colonization assay lacks parasite burden quantification

Lines 299–305:

Please clarify whether parasite load per hindgut was quantified or whether only presence/absence was scored.

8. Speculative interpreatation of osmotic stress effects

Lines 312–320:

The proposed causal link between hyperosmotic stress sensitivity and vector colonization could be phrased more cautiously.

9. TcPDEC absence in metacyclics

Lines 187–190:

Please clarify whether the absense of detectable TcPDEC in metacyclic trypomastigotes reflects downregulation or technical detection limits.

10. TcPDEA and TcPDED not functionally explored

Lines 179–183; Discussion 506–507:

Please briefly justify why these PDEs were not included in functional analyses.

11. Figure legends should include imaging parameters

Figures 1D and 2A–C:

Please provide scale bars, magnification, and acquisition parameters.

12. Statistical reporting

Figures 4–7:

Please indicate exact n values, number of biological replicates, and statistical tests in all figure legnends.

13. Terminology consistency

Throughout Results and Discussion:

Standardize usage of “FT-cAMP,” “flagellar microdomain,” “flagellar tip,” and “flagellum.”

Reviewer #3: It was unclear to me whether the results represent technical triplicates from three different clones (for each transfection) or biological triplicates from a single clone. Please clarify this clearly in the methodology section and in the figure legends.

Lines 234-236: The description of the results should accurately reflect the statistical data presented in Fig. 4D. Although the growth rate decreased compared to the Cas9/T7 control, the AB lines failed to rescue the phenotype when compared to the KO lines, as clearly observed in Fig. 4C.

Figure 4: Please ensure that graphs depicting the same type of experiment use consistent axis scales for proper comparison.

Figure 4F: Please include the statistical analysis comparing PDEB1/2-KO vs. PDEB1-AB and PDEB2-AB.

Lines 257-259: The statement "Together, these results confirm that adhesion is a prerequisite for metacyclogenesis and that FT-cAMP signals, but not CVC-cAMP, mediate both processes in T. cruzi" may be too strong. Based on the data presented, it would be more appropriate to suggest that adhesion correlates with metacyclogenesis, rather than using the term "prerequisite," which implies a stricter causal relationship.

**********

PLOS authors have the option to publish the peer review history of their article (what does this mean?). If published, this will include your full peer review and any attached files.

If you choose “no”, your identity will remain anonymous but your review may still be made public.

**Do you want your identity to be public for this peer review?**  For information about this choice, including consent withdrawal, please see our Privacy Policy.

Reviewer #1: No

Reviewer #2: No

Reviewer #3: No

[NOTE: If reviewer comments were submitted as an attachment file, they will be attached to this email and accessible via the submission site. Please log into your account, locate the manuscript record, and check for the action link "View Attachments". If this link does not appear, there are no attachment files.]

**Figure resubmission:**

-->While revising your submission, we strongly recommend that you use PLOS’s NAAS tool (https://ngplosjournals.pagemajik.ai/artanalysis) to test your figure files. NAAS can convert your figure files to the TIFF file type and meet basic requirements (such as print size, resolution), or provide you with a report on issues that do not meet our requirements and that NAAS cannot fix.

After uploading your figures to PLOS’s NAAS tool - https://ngplosjournals.pagemajik.ai/artanalysis, NAAS will process the files provided and display the results in the "Uploaded Files" section of the page as the processing is complete. If the uploaded figures meet our requirements (or NAAS is able to fix the files to meet our requirements), the figure will be marked as "fixed" above. If NAAS is unable to fix the files, a red "failed" label will appear above. When NAAS has confirmed that the figure files meet our requirements, please download the file via the download option, and include these NAAS processed figure files when submitting your revised manuscript.

**Reproducibility:**

To enhance the reproducibility of your results, we recommend that authors of applicable studies deposit laboratory protocols in protocols.io, where a protocol can be assigned its own identifier (DOI) such that it can be cited independently in the future. Additionally, PLOS ONE offers an option to publish peer-reviewed clinical study protocols. Read more information on sharing protocols at https://plos.org/protocols?utm_medium=editorial-email&utm_source=authorletters&utm_campaign=protocols

# Author comment: Author response to Decision Letter 1

**DOI:** 10.1371/journal.ppat.1013784.r002

4 May 2026

Attachment Submitted filename: Ahmed et al_Response to Reviewers.pdf

# Peer review: Decision Letter 1

**DOI:** 10.1371/journal.ppat.1013784.r003

**Authors:** Jeffrey Dvorin, Tim Nicolai Siegel, Jeffrey Dvorin, Tim Nicolai Siegel

18 Jun 2026

Dear Lander,

We are pleased to inform you that your manuscript 'Cyclic AMP compartmentalization drives signal specificity to control vector colonization and mammalian host infection by American trypanosomes' has been provisionally accepted for publication in PLOS Pathogens.

Before your manuscript can be formally accepted you will need to complete some formatting changes, which you will receive in a follow up email. A member of our team will be in touch with a set of requests.

Please note that your manuscript will not be scheduled for publication until you have made the required changes, so a swift response is appreciated.

IMPORTANT: The editorial review process is now complete. PLOS will only permit corrections to spelling, formatting or significant scientific errors from this point onwards. Requests for major changes, or any which affect the scientific understanding of your work, will cause delays to the publication date of your manuscript.

Should you, your institution's press office or the journal office choose to press release your paper, you will automatically be opted out of early publication. We ask that you notify us now if you or your institution is planning to press release the article. All press must be co-ordinated with PLOS.

Thank you again for supporting Open Access publishing; we are looking forward to publishing your work in PLOS Pathogens.

Best regards,

T. Nicolai Siegel, Ph.D

Academic Editor

PLOS Pathogens

Jeffrey Dvorin

Section Editor

PLOS Pathogens

Sumita Bhaduri-McIntosh

Editor-in-Chief

PLOS Pathogens

orcid.org/0000-0003-2946-9497

Michael Malim

Editor-in-Chief

PLOS Pathogens

orcid.org/0000-0002-7699-2064

***********************************************************

Reviewer Comments (if any, and for reference):

Reviewer's Responses to Questions

**Part I - Summary**

Please use this section to discuss strengths/weaknesses of study, novelty/significance, general execution and scholarship.

Reviewer #1: The authors have made good attempts to revise their manuscript to address the points made in our original review. Although some potential elements of the conclusions or interpretations could be explored further, we think these are sufficiently addressed in the text or will be resolved in future work beyond the scope of this submission.

Reviewer #2: The authors have addressed my previous concerns in a constructive and satisfactory manner. The revised manuscript is considerably improved, particularly in its more cautious treatment of cAMP compartmentalization and the distinction between directly measured total cAMP levels and inferred compartment-specific signaling.

The experimental work remains strong and biologically meaningful. The study provides important insight into how PDE-defined cAMP signaling compartments contribute to T. cruzi differentiation, mammalian host-cell infection, osmoregulation, and vector colonization.

Overall, I consider the revised manuscript suitable for publication in PLOS Pathogens and support acceptance.

Reviewer #3: I thank the authors for considering the criticisms presented in the first round of review. They have adequately clarified the points where there was ambiguity and have satisfactorily made the suggested modifications. The changes to the manuscript have resolved the issues I had identified. I do not identify any new weaknesses that would justify another round of revision. Therefore, I am satisfied with the responses and have nothing further to add.

**********

**Part II – Major Issues: Key Experiments Required for Acceptance**

Please use this section to detail the key new experiments or modifications of existing experiments that should be absolutely required to validate study conclusions.

Generally, there should be no more than 3 such required experiments or major modifications for a "Major Revision" recommendation. If more than 3 experiments are necessary to validate the study conclusions, then you are encouraged to recommend "Reject".

Reviewer #1: (No Response)

Reviewer #2: none

Reviewer #3: (No Response)

**********

**Part III – Minor Issues: Editorial and Data Presentation Modifications**

Please use this section for editorial suggestions as well as relatively minor modifications of existing data that would enhance clarity.

Reviewer #1: (No Response)

Reviewer #2: In a few places, especially in the Abstract, Author Summary, and Discussion, the wording still occasionally remains slightly stronger than the experimental evidence allows. I would encourage the authors to ensure consistent use of cautious terminology such as “PDE-defined cAMP compartments” or “putative cAMP microdomains” throughout the final version. A few minor typographical and grammatical errors should also be corrected during copy-editing.

Reviewer #3: (No Response)

**********

PLOS authors have the option to publish the peer review history of their article (what does this mean?). If published, this will include your full peer review and any attached files.

If you choose “no”, your identity will remain anonymous but your review may still be made public.

**Do you want your identity to be public for this peer review?**  For information about this choice, including consent withdrawal, please see our Privacy Policy.

Reviewer #1: No

Reviewer #2: **Yes:**  Markus Engstler

Reviewer #3: No

# Editor report: Acceptance letter

**DOI:** 10.1371/journal.ppat.1013784.r004

**Authors:** Jeffrey Dvorin, Tim Nicolai Siegel, Jeffrey Dvorin, Tim Nicolai Siegel

Dear Dr. Lander,

We are delighted to inform you that your manuscript, "Cyclic AMP compartmentalization drives signal specificity to control vector colonization and mammalian host infection by American trypanosomes," has been formally accepted for publication in PLOS Pathogens.

We have now passed your article onto the PLOS Production Department who will complete the rest of the pre-publication process. All authors will receive a confirmation email upon publication.

The corresponding author will soon be receiving a typeset proof for review, to ensure errors have not been introduced during production. Please review the PDF proof of your manuscript carefully, as this is the last chance to correct any scientific or type-setting errors. Please note that major changes, or those which affect the scientific understanding of the work, will likely cause delays to the publication date of your manuscript. Note: Proofs for Front Matter articles (Pearls, Reviews, Opinions, etc...) are generated on a different schedule and may not be made available as quickly.

Soon after your final files are uploaded, the early version of your manuscript, if you opted to have an early version of your article, will be published online. The date of the early version will be your article's publication date. The final article will be published to the same URL, and all versions of the paper will be accessible to readers.

For Research Articles, you will receive an invoice from PLOS for your publication fee after your manuscript has reached the completed accept phase. If you receive an email requesting payment before acceptance or for any other service, this may be a phishing scheme. Learn how to identify phishing emails and protect your accounts at https://explore.plos.org/phishing.

Thank you again for supporting open-access publishing; we are looking forward to publishing your work in PLOS Pathogens.

Best regards,

Sumita Bhaduri-McIntosh

Editor-in-Chief

PLOS Pathogens

orcid.org/0000-0003-2946-9497

Michael Malim

Editor-in-Chief

PLOS Pathogens

orcid.org/0000-0002-7699-2064
